# Nanopore sequencing for rapid diagnostics of salmonid RNA viruses

**DOI:** 10.1038/s41598-018-34464-x

**Published:** 2018-11-05

**Authors:** Michael D. Gallagher, Iveta Matejusova, Lien Nguyen, Neil M. Ruane, Knut Falk, Daniel J. Macqueen

**Affiliations:** 1 School of Biological Sciences, University of Aberdeen, Aberdeen, AB24 2TZ United Kingdom; 2 Marine Scotland Science, Marine Laboratory, Aberdeen, AB11 9DB United Kingdom; 3 Fish Health Unit, Marine Institute, Rinville Oranmore, Co, Galway, Ireland; 4 Norwegian Veterinary Institute, Ullevålsveien 68, 0454 Oslo, Norway; 5 Present Address: The Roslin Institute and Royal (Dick) School of Veterinary Studies, The University of Edinburgh, Midlothian, EH25 9RG United Kingdom

## Abstract

Analysis of pathogen genome variation is essential for informing disease management and control measures in farmed animals. For farmed fish, the standard approach is to use PCR and Sanger sequencing to study partial regions of pathogen genomes, with second and third-generation sequencing tools yet to be widely applied. Here we demonstrate rapid and accurate sequencing of two disease-causing viruses affecting global salmonid aquaculture, salmonid alphavirus (SAV) and infectious salmon anaemia virus (ISAV), using third-generation nanopore sequencing on the MinION platform (Oxford Nanopore Technologies). Our approach complements PCR from infected material with MinION sequencing to recover genomic information that matches near perfectly to Sanger-verified references. We use this method to present the first SAV subtype-6 genome, which branches as the sister to all other SAV lineages in a genome-wide phylogenetic reconstruction. MinION sequencing offers an effective strategy for fast, genome-wide analysis of fish viruses, with major potential applications for diagnostics and robust investigations into the origins and spread of disease outbreaks.

## Introduction

Pathogen genome sequencing greatly enhances the study of viral disease evolution, phylogeography and epidemiology^1^, including human epidemics such as Ebola^2^, HIV^3^, and influenza^4,5^. Second-generation sequencing platforms (e.g. Illumina) are now used routinely for genome-wide monitoring and investigations of viral disease, and generate accurate short-read data at massive throughput^6–8^, typically requiring computationally-intensive analysis pipelines. Third-generation platforms, including single-molecule real time (SMRT)^9^ and Oxford Nanopore^10^ show high promise for genome-wide analysis of viruses^11,12^, and bring the additional benefit of longer sequencing reads offset by higher error rates. The MinION nanopore sequencer is a particularly promising technology for viral research and diagnostics, owing to several unique features (i.e. portability, low start-up costs, real-time data generation and straightforward application) that have, for example, allowed human pathogens to be rapidly characterized in the field without high-power computing or major laboratory infrastructure^13,14^.

Aquaculture is the fastest growing food production sector^15^, yet its sustainability and expansion is threatened by infectious diseases. Among a list of concerning pathogens, several known viral disease agents cause major animal health and welfare issues, accompanied by massive financial losses through mortalities, slow growth, poor flesh quality, treatment interventions and control protocols (e.g. culling)^16,17^. Accurate diagnosis of viral diseases is an essential part of strategic planning to manage existing and limit future outbreaks, and is especially important considering the lack of fully-effective treatments and vaccines for most fish viral pathogens (e.g.^18–20^). Recommended diagnostic procedures of viral disease include demonstration of clinical pathology coupled to the presence of pathogen DNA/RNA, followed by culturing to establish the presence of viable pathogen^21^. Diagnostic sequencing of aquatic viruses is typically done by PCR and Sanger sequencing, which benefits from high accuracy and established protocols. However, such approaches are limited to relatively short sequences (i.e. up to 1500 bp when sequencing both directions) and cannot gain a genome-wide representation of viruses and their variants without non-routine effort. Second and third generation sequencing tools hold promise for the characterization of aquatic viruses (reviewed in^22,23^), including pathogens affecting global fish aquaculture, yet they are being up-taken relatively slowly. The utility of such approaches have been demonstrated by the characterisation of novel pathogens such as Tilapia Lake Virus (TiLV) using Ion Torrent sequencing^24^, the discovery of Piscine Reovirus (PRV)^25^ and Piscine myocarditis virus (PMCV)^26^ with pyrosequencing, and the analysis of Cyprinid herpesvirus 3 genomes using a target enrichment and Illumina sequencing approach to identify mixed genotype infections^27^. However, as far as we are aware, to date no published studies have successfully used MinION sequencing to study viral diseases impacting farmed fish.

In this study, we demonstrate rapid genome-wide sequencing of fish viral pathogens using nanopore sequencing on the MinION platform. We focussed on two disease agents affecting farmed Atlantic salmon (*Salmo salar* L.), salmonid alphavirus (SAV) and infectious salmon anaemia virus (ISAV). SAV is a single-strand positive-strand RNA virus (Family *Togaviridae*) and the causative agent of pancreas disease, prevalent across European salmon aquaculture, with six SAV subtypes (SAV1-6) established^28^. All SAV sequences published to date have been generated using the Sanger method, including full genomes for SAV1-3^29–33^, and partial genomic regions primarily encoding a glycoprotein (E2) or a non-structural protein (NsP3) (neither representing known virulence markers), for samples representing all six subtypes (e.g.^28,34^). ISAV is a highly pathogenic, segmented, negative-strand RNA virus (Family *Orthomyxoviridae*) often resulting in high mortality rates^35,36^, with containment and culling being the only effective mitigation strategy^37^. ISAV genomes have been Sanger-sequenced from several ‘genogroups’^38–43^, while segments 5 and 6, which contain known virulence markers and respectively encode the fusion and hemagglutinin surface proteins, are routinely used for Sanger genotyping, but have also been characterized using Illumina sequencing^44^. Overall, in common with other fish viruses, there is a lack of genome-wide data for SAV and ISAV, limiting power to define virulence markers and understand the evolution of different viral lineages. This study linked MinION sequencing to standard PCR enrichment to accurately sequence and genotype both SAV and ISAV. In addition to reporting the first full genome sequence for SAV6, we discuss the potentially transformative applications of MinION sequencing in diagnostics and molecular epidemiology of viruses impacting aquaculture.

## Results and Discussion

### SAV genome-wide sequencing

Using primers matching conserved regions of the SAV genome (Table 1), three overlapping PCR amplicons (approx. 4 kb each; Supplementary Fig. 1) were obtained from two samples known to represent SAV1 (SCO/4640/08) and SAV6 (F1045-96) (Table 2) and sequenced on separate MinION flow cells (R9.4) for 2–3 hours (sequencing details provided in Supplementary Table 1). Over 98% of each SAV genome was recovered with 90 bp missing at the 5′ and 30 bp at the 3′ region of the genome due to the location of the highly conserved primer binding sites. The average read length from both sequencing runs was ~3800 bp per amplicon, indicating limited DNA shearing during the library preparation. The sequencing of sample SCO/4640/08 was stopped after 3-hours producing over 400 Mb of ‘pass’ reads (Q-score ≥ 7), resulting in almost 40,000x coverage throughout the genome (Table 3). By mapping against the Sanger sequenced SAV1 reference sequence for SCO/4640/08^32^

**Table 1:** PCR primers used in the study.

Virus	Primer Name	Primer sequence/5′ - 3′	Amplicon Length
SAV	Structural	CMAACTCAGCCTAYCGCCAG	3914
		GCACTTCTTCACCACGCAG	
	Non-structural/a	AGACTGCGTTTCCAGGGTT	4078
		ATGTCGGTCAGTTGAGGGC	
	Non-structural/b	AGTGGGAYWCTAAGCCGAGAGG	4488
		TACACGGGGAAGGTGCTCTG	
ISAV	Fusion	ATGGCTTTTCTAACAATTTTAGTCT	1301
		AGCACCACCAACACAACTACA	
	Hemagglutinin	GGCACGATTCATAATTTTATTCCT	1146*
		GAACAGAGCAATCCCAAAACCT	

Primers were designed in conserved regions of the genome to be applicable for a wide range of strains.

*The amplicon length of an isolate with a full HPR region/HPR0.

**Table 2:** Details of isolates used for MinION sequencing.

Virus	Isolate	Year of Isolation	Country of Isolation	Cell Line	Subtype
SAV	SCO/4640/08	2008	United Kingdom	CHSE	SAV1
	F1045-96	1996	Ireland	BF2/EPC	SAV6
ISAV	SCO/4750/09	2009	United Kingdom	NA	EU-G1
	CA/NB04-85-1/04	2004	Canada	NA	EU-NA
	CA/NB7178/08	2008	Canada	NA	EU-NA
	CA/F679/99	1999	Canada	NA	EU-NA
	NO/Sotra/B797/92	1992	Norway	NA	EU-G3
	SCO/4661/08	2008	United Kingdom	NA	EU-G1
	NO/Glessvær/2/90	1990	Norway	NA	EU-G2

**Table 3:** MinION sequencing details after basecalling and quality control.

Virus	Isolate	No. of Reads sequenced	No. of reads mapped	% reads mapped	Average Genome Coverage	ISAV HPR
SAV	F1045-96	73,574	66,705	91	21,306	—
	SCO/4640/08	112,805	93,998	83	39,012	—
ISAV	SCO/4750/09	25,009	24,797	99	9,609	HPR35
	CA/NB04-85-1/04	11,816	11,201	95	9,932	Uncharacterised
	CA/NB7178/08	20,136	19,672	98	4,464	Uncharacterised
	CA/F679/99	18,650	18,308	98	4,593	Uncharacterised
	NO/Sotra/B797/92	13,410	13,192	98	4,950	HPR1
	SCO/4661/08	23,793	23,584	99	9,710	HPR35
	NO/Glessvær/2/90	39,343	38,463	98	19,232	HPR2

ISAV HPR classification based on established genotypes outlined in Nylund *et al*. 2003.

As the above approach led to an accurate representation of a verified SAV genome sequence, we can be confident in its application to discovering entirely novel variation. For this reason, we decided to sequence SAV6 (sample F1045-96), which has only been identified once, as partial E2 and NsP3 sequences, from a single Irish sample^28^, and is highly distinct from all other subtypes. After two hours of sequencing, a genome-wide average of 21,000x coverage was achieved. The SAV6 genome consensus showed 100% similarity to Sanger-sequenced NsP3 (EF675499) and E2 (EF675547) gene sequences. Table 4 shows consistent genome-wide pairwise similarities contrasting the genome of SAV6 to the other SAV sub-types at both nucleotide and amino acid level (88.6–89.2% and 93.8–94.6% respectively). Variability among SAV subtypes differed based on the gene of interest and the greatest variability was seen in the NsP3 gene (82.0–83.8% and 87.7–89.8% nucleotide/amino acid similarity). In conclusion, these data gained by MinION sequencing confirm for the first time using genome-wide evidence that SAV6 represents a highly-divergent SAV subtype.

**Table 4:** Pairwise similarities between SAV6 and reference genomes for SAV1-5.

Gene	SAV6/SAV1 (NUC/AA)	SAV6/SAV2 (NUC/AA)	SAV6/SAV3 (NUC/AA)	SAV6/SAV4 (NUC/AA)	SAV6/SAV5 (NUC/AA)
NSP1	91.8/94.7	91.6/95.5	92.7/95.7	91.8/95.3	92.3/95.7
NSP2	89.9/95.6	89.8/95.9	89.8/96.7	89.5/95.8	89.8/96.2
NSP3	83.8/88.9	82.8/88.2	82.0/87.7	83.2/89.4	83.8/89.8
NSP4	87.9/95.6	89.3/96.1	88.5/96.4	87.5/95.2	88.3/96.2
CP	90.2/91.8	89.2/90.8	90.3/93.3	90.7/93.3	91.5/92.9
E3	88.7/93.0	85.4/93.0	86.9/94.4	83.6/88.7	85.9/93.0
E2	87.8/92.7	87.1/91.8	87.2/92.9	85.5/92.5	87.0/93.4
6K	91.2/95.6	89.7/94.1	93.1/97.1	91.7/97.1	91.2/95.6
E1	91.4/96.7	90.5/96.2	90.6/95.6	90.9/96.9	91.9/97.1
Genome	89.2/93.9	88.6/93.8	88.7/94.3	88.3/94.2	89.0/94.6

Reference sequences as follows: SAV1/AJ316244, SAV 2/AJ316246 and SAV3/KC122925.

References for SAV4 (SAV 04-44) and SAV5 (SAV SCO10-684) were generated in this study.

### Genome-wide SAV phylogeny

**Figure 1:**
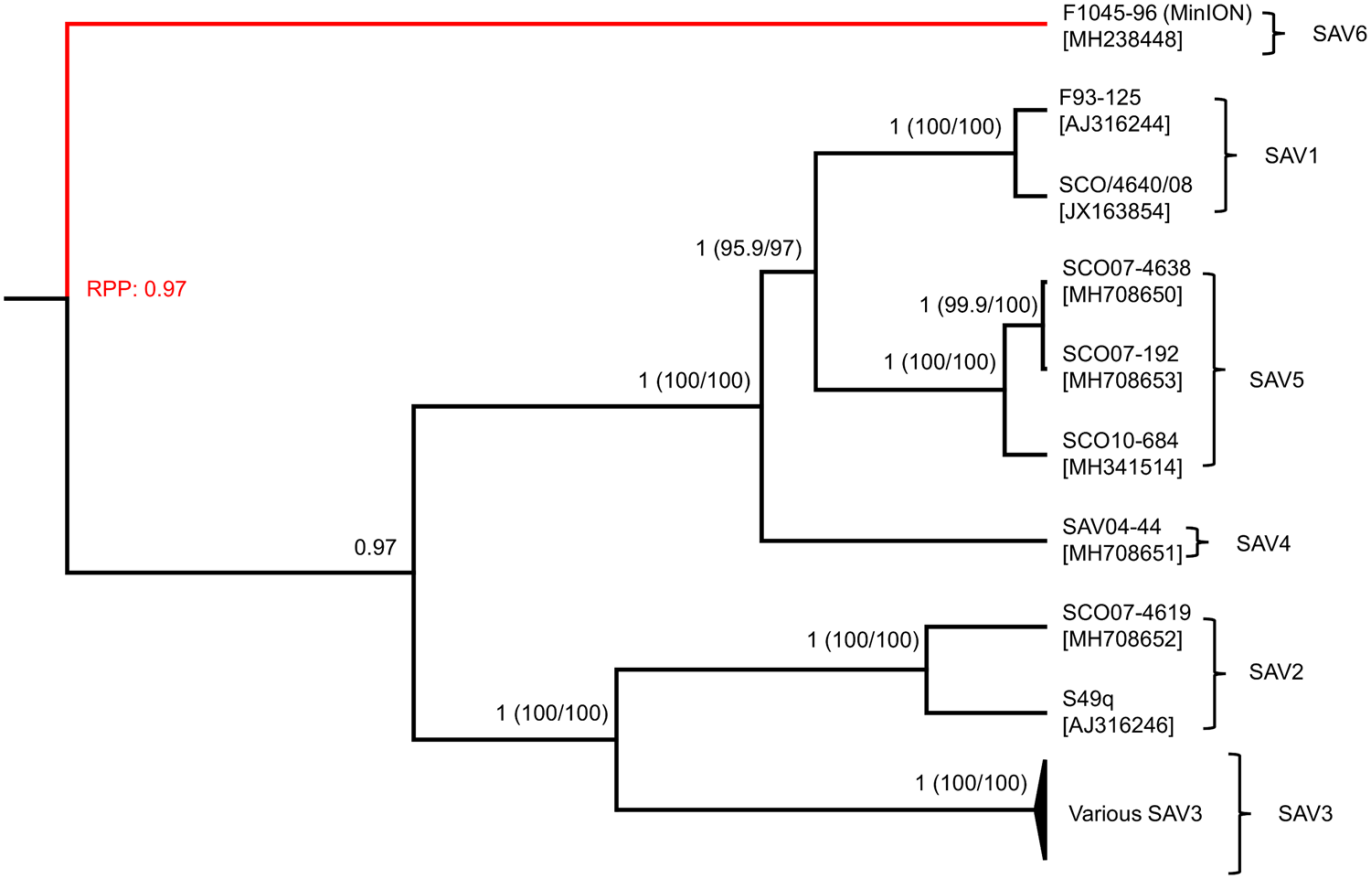
Genome-wide Bayesian phylogeny for SAV lineages including the SAV6 sequence generated by MinION sequencing (shown in red). The data represents an 11,638 bp nucleotide alignment and the analysis was done using the best-fitting nucleotide substitution model (GTR) and a relaxed molecular clock model. Branch posterior probability support is shown with comparable ML bootstrap support given in parentheses. Branch lengths are scaled in relative time as the relaxed clock model was uncalibrated. Root posterior probability (RPP) estimated by RootAnnotator^46^ is shown in red font.

Previous studies have failed to establish the position of SAV6 within the SAV phylogeny based on E2 and NsP3 sequences (e.g.^28,34^). We performed genome-wide phylogenetic reconstructions incorporating the new SAV6 genome gained by MinION sequencing, along with 17 SAV genomes available in NCBI, and 5 new (i.e. previously unpublished) Sanger-sequenced genomes for SAV2, 4 and 5 (isolate details in Supplementary Table 2). We used two probabilistic methods, the first a Bayesian approach incorporating a relaxed clock model^45^ allowing estimation of the tree root^46^ and the second an unrooted maximum-likelihood (ML) approach (Fig. 1). The root of the SAV phylogeny was estimated with high confidence (posterior probability: 0.97), and split SAV6 from all other SAV sub-types. Branching of other subtypes was maximally supported (posterior probability: 1.0; ML bootstrap values > 95%), with SAV3 and 2 forming a monophyletic group separate from a clade containing SAV1, 4 and 5 (Fig. 1). The basal phylogenetic position of SAV6 highlights particular importance for the new MinION genome sequence in future investigations of the evolution and phylogeography of the major SAV lineages.

### ISAV segment 5 and 6 sequencing

To test MinION sequencing on a distinct fish virus, we focused our efforts on ISAV, which exists in eight genomic segments (length: 740–2169 bp). This inherent aspect of the virus limits one of the main benefits of MinION sequencing: its capacity to generate genome-wide representation of a virus with a small number of overlapping PCR amplicons, as done successfully for SAV. We instead focused on ISAV segments 5 and 6, which are widely studied and known to contain ISAV virulence markers, this time testing a barcoding approach to sequence multiple samples on a single MinION flow cell. PCR amplicons (primers in Table 1) amplifying 97% of segment 5 and 93% of segment 6 including both virulence markers, were obtained from seven ISAV isolates (Table 2) and pooled in equimolar amounts for sequencing after barcoding. After 3 hours, approx. 9,000x mean coverage was achieved per sample. Only one of the isolates used in this study has a reference Sanger sequence (NO/Glessvær/2/90); basecalling accuracy was estimated for segments 5 and 6 of this isolate and 100% similarity was observed.

ISAV segment 6 contains a highly polymorphic region (HPR) at the 3′ end of the gene which is a known virulence marker. The putatively non-pathogenic ISAV, called HPR0, is characterized by a full length of the HPR comprising 35 amino acids and all pathogenic ISAV strains to date (called HPR-deleted) contain a deletion in the HPR region of varying length^47^. While none of the isolates used in this study were HPR0, the HPR of all the ISAV isolates used in this study were successfully classified with several different deletions being identified including three samples CA/NB04-85-1/04, CA/NB7178/08, CA/F679/99 which have a deletion previously found only once before and not yet fully characterised^48^ (Table 3). In addition, the consensus sequences for each segment 5 captured another proposed virulence marker, the substitution Q_266_L^39,49,50^, with all but one isolate (CA/NB04-85-1/04) possessing the L variant. CA/NB04-85-1/04 instead encodes for a proline at this position which while unusual, is also present in a Canadian isolate from the EU/NA genogroup (EF432567)^51^. These data thus demonstrate that MinION sequencing effectively recaptures sequence-level virulence markers.

### Optimal sequence coverage

**Figure 2:**
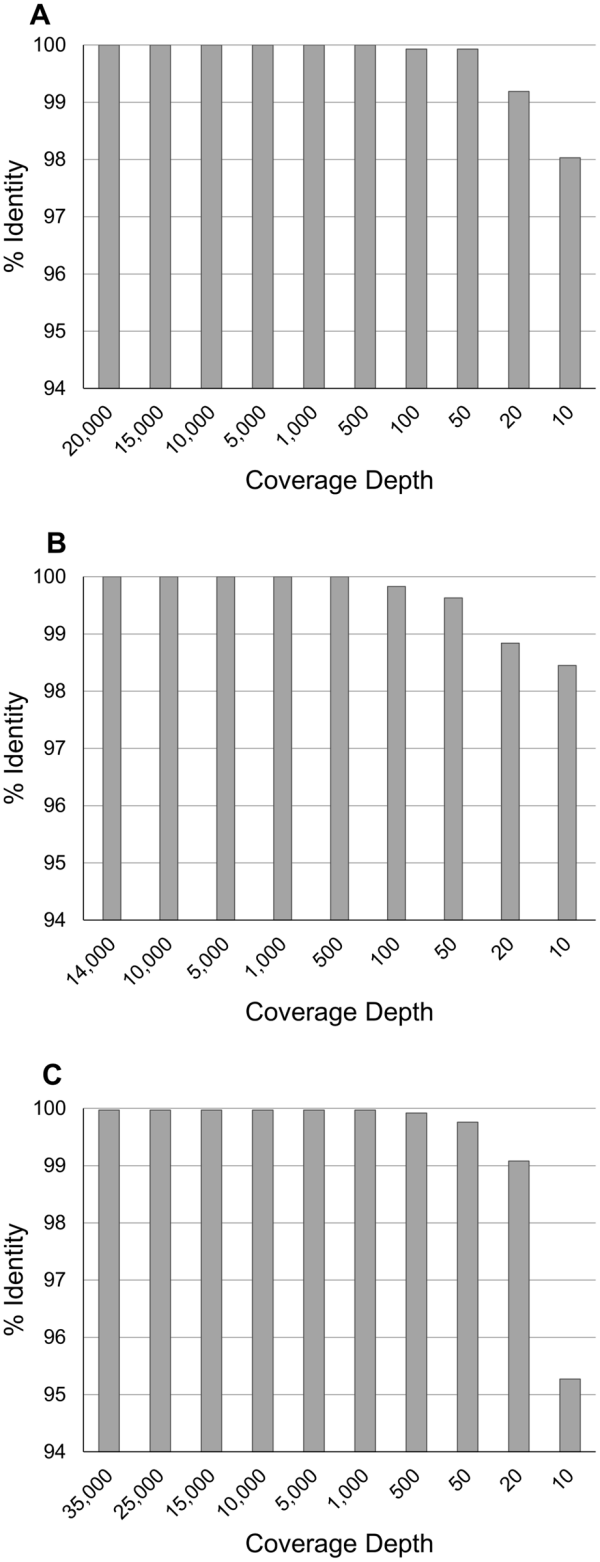
Impact of MinION read coverage on accuracy of consensus sequence generation. ‘% identity’ is shown between reference Sanger sequences and consensus sequences generated from randomly sampling MinION reads at multiple sequence coverages for: (**A**) Segment 5 of ISAV NO/Glessvær/2/90; (**B**) Segment 6 of ISAV NO/Glessvær/2/90; (**C**) SAV1 genome (sample SCO/4640/08).

Future studies would benefit from establishing the necessary coverage required to determine confident consensus sequences using MinION. Thus, we randomly sampled MinION reads mapping to segments 5 and 6 of one ISAV sample (NO/Glessvær/2/90) and the SAV1 genome (sample: SCO/4640/08) at different coverages to establish the impact on consensus sequence accuracy (Fig. 2A–C). 50x and 500x coverage of either ISAV segment achieved a consensus sequence >99% and 100% identical to the Sanger reference, respectively (Fig. 2A,B). For SAV1, just 20x coverage led to 99% similarity with the Sanger reference, while 1,000x coverage led to 99.97% similarity (Fig. 2C). Thus, despite its high error rate (e.g.^10^), a highly-accurate consensus sequence can be generated with very modest MinION sequencing time.

### Broader perspectives and comparisons with other platforms

Rapid sequencing of two structurally-distinct fish RNA viruses was achieved with high accuracy using MinION sequencing coupled with PCR. While the samples used were from cultured viruses, we have had equal success using the same protocols and infected tissues with much lower virus titres (data not shown). The methods described were achieved within 24 hours lab-time, exploiting PCR primers matching conserved genomic regions, which allowed a highly divergent viral genome (SAV6) to be sequenced with little prior knowledge of sequence variation. Combining such turn-around and ease of application with the accuracy gained from moderate sequencing coverage opens the doorway to routine high-confidence viral genotyping at shallow phylogenetic scales, sufficient for robust diagnostics supporting disease management and regulatory decisions. Elsewhere, it has also been shown that MinION sequencing can be used to recover viral RNA genomes from infected samples without prior PCR enrichment, which has advantages in the field^52^ and can also potentially identify viruses beyond the target pathogen. The ease of generating genome-wide sequencing data for non-segmented viruses such as SAV has revolutionary potential for diversifying the relatively restricted current repertoire of publicly-available fish virus genomes, bringing benefits for fundamental research and disease management. However, it is important to acknowledge that our approach is best-suited to generating consensus viral genome sequences, and less useful for identifying population variation within samples, which is well-established for RNA viruses^53–58^, as the PCR enrichment may introduce biases toward particular variants, and the high sequencing error rate of MinION reduces power to call low frequency variants *de novo*.

Future efforts should also aim to reduce the cost of genome-wide sequencing using multiplexing to exploit the high coverage possible on a single MinION flow cell. We estimate that the single SAV genomes (~12 kb) generated in our study cost approx. £850 each, including all consumables and an entire flow cell; however, multiplexing using 96 samples and the same approach would reduce this cost to approx. £50–60 per sample. By comparison, it would not be possible to perform a direct-equivalent Sanger sequencing approach, as the amplicon length exceeds the possible length of sequenced reads. Assuming an SAV genome was tiled across 7 PCR amplicons (e.g.^32^) and sequenced directly using Sanger with no cloning step (which would add further costs), we estimate a cost of approx. £100 per SAV consensus genome, including all reagents and bi-directional sequencing. In addition to a per-genome saving, the MinION approach is more convenient and time-efficient when a large number of genomes need to be sequenced, being done in-house in a single sequencing run with fewer amplicons, avoiding the need for cloning and the use of an external Sanger provider. It is more challenging to directly compare costs of our MinION strategy with alternative high-throughput approaches, as there are many platforms and variations in library preparation strategy, and this would also be affected by the extent of sub-contracting to an external provider. However, we estimate that the costs of generating complete SAV genomes using Illumina at the same scale (i.e. 96 samples), assuming the same amplicon strategy followed by in-house library preparation/indexing (Nextera XT DNA kit) and sequencing on the MiSeq platform by an external provider to be approx. £50–65 (i.e. very comparable). While Illumina brings advantages in terms of data accuracy, e.g. giving more scope for detecting viral population variation, the MinION avoids use of an external provider, which typically leads to a lag of weeks to months for delivery. Overall, our MinION approach has some cost and/or time advantages when compared to Sanger and Illumina approaches if the aim is to recover a consensus SAV genome with high accuracy, and future work is needed to develop this approach for robust analysis of viral population variation.

In conclusion, once low cost MinION sequencing of fish viral genomes is achieved, considering the unique portability of the sequencer alongside the modest computational power needed to analyse the resultant data, it seems reasonable to anticipate in-field diagnostic applications in the near future, including the monitoring of viral genotypes and subtypes directly on fish farms and in the field.

## Materials and Methods

### Sample preparation and PCR

Total RNA was extracted from SAV and ISAV samples (Table 2) using a phenol-chloroform extraction method, except for the SAV6 sample, which was extracted using a Viral RNA Isolation kit (Qiagen). cDNA was synthesised using Protoscript II (New England Biolabs) reverse transcriptase and a mix of random hexamer and oligo dT (dT_23_VN) primers (New England Biolabs) as per the manufacturers’ instructions. First-strand cDNA was used as template for long-range PCR reactions.

To amplify the SAV1/6 genomes, degenerate PCR primers targeting three ~4 kb overlapping amplicons were designed in regions of the genome conserved in the five subtypes where sequence data is available (Table 1). PCR was conducted using LongAmp polymerase (New England Biolabs) with cycling conditions as follows: 30 s at 94 °C, followed by 35 cycles of 15 s at 94 °C, 1 min at 56 °C and 3 min 50 s at 65 °C, with a final extension for 10 min at 65 °C. ISAV segments 5 and 6 were amplified using the same approach and primers designed to conserved 5′ and 3′ regions of segment 5/6 (Table 2) under the same conditions, except that the PCR extension time was 2 min 30 s. PCR products were visualised on a 1% agarose gel, purified using QIAquick Gel Extraction Kit (Qiagen) and stored at −80 °C until sequencing.

### Sanger sequencing of novel SAV genomes

Seven overlapping PCRs were performed in triplicates for five SAV isolates (Supplementary Table 2) according to the methods published by Matejusova *et al*.^32^. The complete SAV genomes were generated by Sanger sequencing, assembled using Sequencher v5.4.6 and used in the phylogenetic analysis presented in Fig. 1.

### Preparation of SAV Library and sequencing

1000 ng of equimolar pooled amplicon from each SAV isolate was the input to a library generated with the Ligation Sequencing Kit 1D SQK-LSK108 (Oxford Nanopore Technologies). Before ligating sequencing adaptors, DNA was end-repaired using the NEBNext Ultra II End Repair/dA Tailing kit (New England Biolabs), purified using AMPure XP beads (Beckman Coulter) in a ratio of 1:1 volume of beads per sample and eluted in 30 µl of nuclease-free water (Sigma). Sequencing adapters (AMX1D) (ONT) were ligated to the DNA using Blunt/TA Ligation Master Mix (New England Biolabs) by incubation at room temperature for 10 min. The adapter-ligated DNA library was purified with AMPure XP beads in a ratio of 1:2.5 volume of beads per sample, followed by a wash with Adapter Bead Binding buffer (ABB) (ONT) and elution in 15 µl nuclease-free water. DNA concentrations were determined between each step using a Qubit fluorimeter (Fisher Thermo). Each cleaned library was loaded onto a separate MinION Flow Cell Mk1 R9.4 (ONT) and run via MinKNOW software (without real-time basecalling) for 2 and 3 hours for SAV6 (F1045-96) and SAV1 (SCO/4640/08) respectively.

### Preparation of ISAV Library and Sequencing

The ISAV library was prepared using the Ligation Sequencing Kit 1D SQK-LSK108 and a Native Barcoding Kit EXP-NBD103 (Oxford Nanopore Technologies). Segments 5 and 6 from the same virus isolate were pooled in equimolar amounts and 300 ng of each isolate end-repaired using the NEBNext Ultra II End Repair/dA Tailing kit. DNA was purified using AMPure XP beads in a ratio of 1:1 volume of beads per sample and eluted in 30 µl nuclease-free water. Native barcodes were ligated to 200 ng of end-repaired DNA using Blunt/TA Ligation Master Mix. The barcoded DNA was purified using AMPure XP beads in a ratio of 1:1 volume of beads to sample to remove excess barcodes and eluted in 26 µl nuclease-free water. The barcoded samples were pooled in equimolar amounts to a total of 200 ng library DNA (~0.2 pmol as per Oxford Nanopore Technologies instructions). Barcode adapter mix (BAM) (ONT) was ligated to the library DNA using NEBNext Quick Ligation Reaction Buffer and Quick T4 DNA Ligase (New England Biolabs), and incubated at room temperature for 10 min. Library DNA was purified using AMPure XP beads in a ratio of 1:2.5 volume of beads per sample and subsequently washed with Adapter Bead Binding buffer (ABB) before elution in 15 µl nuclease-free water. DNA concentrations were determined between each step as above. Libraries were loaded according to the native barcoding kit protocol (ONT) onto a MinION Flow Cell Mk1 R9.5., using a 3-hour sequencing run via MinKNOW without real-time basecalling.

### Basecalling and consensus assembly

MinION data basecalling and demultiplexing for barcoded ISAV samples was performed using Albacore v.2.1.7 on Windows command line. Base-called FASTQ files were loaded into Geneious v.10^59^ for mapping and analysis. SAV1 (SCO/4640/08) sample reads were mapped to the SAV1 Sanger-reference sequence^32^. SAV6 (F1045-96) sample reads were individually mapped to the partial gene E2 and NsP3 sequences of SAV6^28^ and reference genomes for SAV1^32^, SAV2^29^, SAV3^33^, SAV4 (generated in this study; isolate SAV 04-44) and SAV5 (generated in this study; isolate SCO10-684). In order to reconstruct the whole SAV6 genome, mapping was set at 5 iterations and a 65% consensus threshold. The 5 generated SAV consensus sequences were then manually inspected and any single base ambiguities resolved by parsimony, giving a final F1045-96 (SAV6) consensus sequence. For example, at position 2235, 4 out of 5 consensus sequences were the base G, whereas one consensus sequence was A: in this case, G was adopted for the final consensus. The ISAV samples were individually mapped to the previously sequenced segment 5 and 6 of the Scot157/08 isolate^60^ using the same parameters.

Reads for ISAV NO/Glessvær/2/90 segments 5 and 6, and SAV1 (SCO/4640/08) were subjected to random subsampling to determine the depth of coverage necessary to generate an accurate consensus (i.e. Fig. 2). Subsampling was performed in Geneious v.10 using the ‘Randomly Sample Sequences’ workflow. Subsampled reads were realigned to the reference sequences using the same mapping methods as above and consensus sequences were generated from each alignment and compared to the reference Sanger sequence using pairwise alignment. Consensus sequences were aligned against all published genome sequences using MAFFT v.7^61^ and manually inspected for errors in the mapping that disrupted the protein coding sequences in BioEdit software v.7.2.5^62^. Sequence pairwise similarities were calculated using Geneious statistics of the MAFFT-aligned whole genome sequences.

### Genome-wide SAV phylogenetic analyses

Multiple sequence alignment of 23 SAV genomes (Supplementary Table 2) was done using MAFFT v.7, generating an 11,638 bp alignment (provided in Supplementary Dataset 1), which was uploaded to the IQ-TREE server^63^ to determine the best-fitting nucleotide substitution model (GTR) and generate a phylogenetic tree with support values gained from 1,000 Ultrafast Bootstrap iterations^64^. Bayesian phylogenetic analysis was done using the same dataset in BEAST2^65^ employing a relaxed clock model^45^, a Coalescent Bayesian Skyline tree model^66^, the GTR substitution model and a Markov Chain Monte Carlo (MCMC) chain of 200 million generations. Tracer^67^ was used to assess MCMC convergence and estimate effective sample sizes for all sampled parameters (>2,000 in all cases). TreeAnnotator was used to remove the first 10% of sampled trees as burn-in and produce a Maximum Credibility Clade (MCC) tree. RootAnnotator^46^ was used to estimate posterior support for alternative root positions. MCC trees were visualized using FigTree (http://tree.bio.ed.ac.uk/software/figtree/).

## Electronic supplementary material

Dataset 1

Supplementary Information

## Data Availability

MinION sequences for SAV isolates: SRA BioProject Accession SRP142226. SAV6 consensus genome: NCBI accession MH238448. MinION sequences for ISAV: SRA BioProject Accession SRP155694. ISAV segments 5 and 6: NCBI accessions: MH708654-MH708667. Sanger-sequenced SAV genomes: NCBI accessions MH341514 and MH708650-MH708653.
