# Supplementary material for: Nanopore sequencing for rapid diagnostics of salmonid RNA viruses
Source: Sci Rep. 2018 Nov 5;8:16307. doi: 10.1038/s41598-018-34464-x (PMC6218516; doi:10.1038/s41598-018-34464-x)
Supplement: Supplementary file 2 — Supplementary Information [file 41598_2018_34464_MOESM2_ESM.pdf]

Supplementary Information

Nanopore sequencing for rapid diagnostics of salmonid RNA viruses

Michael D. Gallagher, Iveta Matejusova, Lien Nguyen, Neil M. Ruane, Knut Falk, Daniel J. Macqueen

Includes:

- Supplementary Table 1
- Supplementary Table 2
- Supplementary Figure 1

Supplementary Table 1. Information on the MinION sample runs achieved in the study (determined by Albacore basecalling output)

Sample	Active Channels	Mean Read Length	Mean Read Quality	Median Read Length	Median Read Quality	Number of reads	Read length N50	Total Bases	# Reads > q-score 7	% Reads > q-score 7	# Bases > q-score 7	Hours Run
ISAV	511	1,100	9.7	1,212	10.5	246,723	1,240	271,313,213	212,734	86	258,300,000	3
SAV1	294	3,875	9.4	3,902	10.1	112,815	4,243	252,632,943	73,488	82	229,895,978	3
SAV6	339	3,920	10.6	3,994	11.0	80,490	4,027	315,477,290	76,960	96	307,100,000	2

Supplementary Table 2. SAV genome isolate details

Virus Strain	Year	Country of Origin	Subtype	Accession Number
F93-125	1993	Ireland	SAV1	AJ316244
S49q	1995	France	SAV2	AJ316246
SavH20/03	2003	Norway	SAV3	AY604235
SAVH10/02	2002	Norway	SAV3	AY604236
PD97-N3	1997	Norway	SAV3	AY604237
SavSF21/03	2003	Norway	SAV3	AY604238
H10	2007	Norway	SAV3	JQ799139
SCO/4640/08	2008	United Kingdom	SAV1	JX163854
SAV3-7-R/09	2009	Norway	SAV3	KC122918
SAV3-9-R/10	2010	Norway	SAV3	KC122919
SAV3-5-H/10	2010	Norway	SAV3	KC122920
SAV3-8-R/10	2010	Norway	SAV3	KC122921
SAV3-6-H/10	2010	Norway	SAV3	KC122922
SAV3-4-SF/10	2010	Norway	SAV3	KC122923
SAV3-1-T/10	2010	Norway	SAV3	KC122924
SAV3-3-MR/10	2010	Norway	SAV3	KC122925
SAV3-2-MR/10	2010	Norway	SAV3	KC122926
SAV SCO07-4619	2007	United Kingdom	SAV2	MH708652
SAV 04-44	2004	Ireland	SAV4	MH708651
SAV SCO10-684	2010	United Kingdom	SAV5	MH341514
SAV SCO07-4638	2007	United Kingdom	SAV5	MH708650
SAV SCO07-192	2007	Scotland	SAV5	MH708653
F1045-96	1996	Ireland	SAV6	MH238448

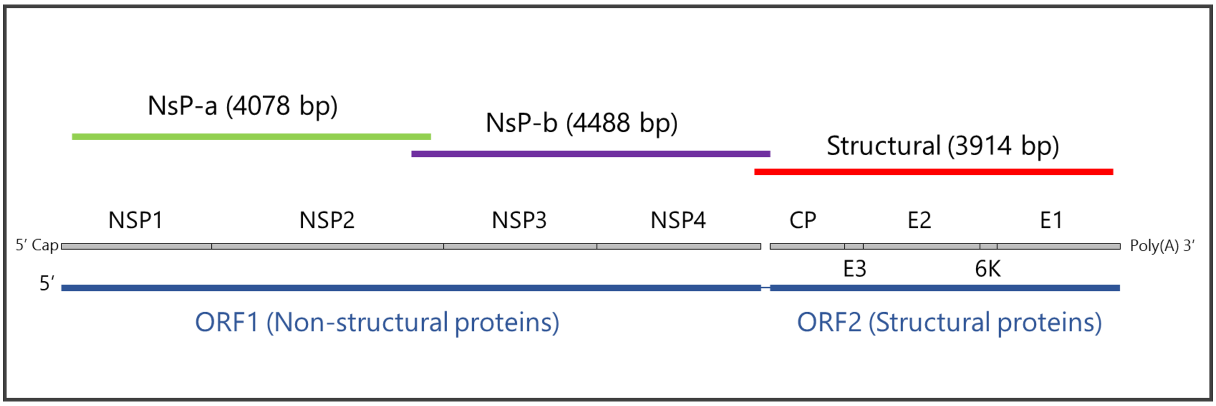

Supplementary Figure 1. Schematic of the three overlapping PCR amplicons covering >98% of the SAV genome. Amplicons are coloured on top (NsP-a, NsP-b, Structural) with corresponding genomic locations to scale in grey and blue.
